# Acid Phosphatase Produced by *Trichoderma harzianum* in Solid Fermentation Using Millet

**DOI:** 10.1021/acsomega.4c10512

**Published:** 2025-07-09

**Authors:** Jussara Maria Martins de Almeida Afonso, Frederico Alves Lima, Miriam Maria de Resende

**Affiliations:** Chemical Engineering Faculty, Federal University of Uberlândia, P.O. Box 593, Av. João Naves de Ávila 2121, Campus Santa Mônica, Bloco 1K, 38408-144 Uberlândia, MG, Brazil

## Abstract

The absorption of organic phosphorus from the environment and its efficient use requires the action of a large class of enzymes called acid phosphatases. Fungi can convert insoluble phosphorus into soluble forms during the acidification and chelation stages or through hydrolysis mediated by phosphatase enzymes. These enzymes are obtained from plants, animal cells, and microorganisms. Acid phosphatases (AcPases) have their optimum pH of action being less than 6.00. This study evaluated the production and extraction of acid phosphatases using solid fermentation (FES) with *Trichoderma harzianum* supported in millet. Preliminary tests were fundamental in analyzing the enzyme production potential of the *T. harzianum* strain, where the best results were for millet. Using a Central Composite Design (CCD), it was possible to develop equations for responses to acid phosphatase activity (U/mL) and dry biomass (g/L) using the variables: millet mass, inoculum concentration, and moisture. The best results were for solid fermentation, using the millet substrate with cultivation in sterile distilled water and extraction with Tween 80, where there was a growth of biomass and enzymatic activity, whose optimized final values were 9.27 ± 0.53 g/L and 36.09 ± 0.61 U/mL, respectively, after 9 days of solid fermentation.

## Graphical abstract

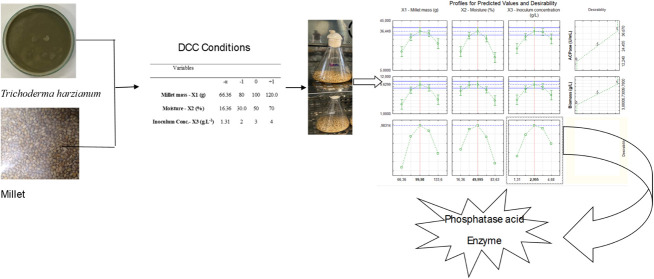

## 1 Introduction

Phosphorus (P) is one of the major plant nutrients, and its deficiency substantially reduces plant growth and production. P is involved in many physiological processes of plants like photosynthesis, energy transfer, and synthesis of antioxidants.^1^ In agricultural practices, millions of tons of phosphatic fertilizers are intentionally applied to soils each year for plant growth. To be the most effective, efficient, and environmentally favorable, however, the entire applied mass of the phosphatic fertilizers should remain available to plants near the root zone.^2^

Phosphatases are a group of enzymes that catalyze the hydrolysis of a phosphate ester compound to release inorganic phosphate (IP). Depending on their optimum pH for their catalytic mechanism, phosphatases are classified into two distinct categories: Alkaline phosphatase (ALP, E.C. 3.1.3.1) and acid phosphatase (ACP, E.C. 3.1.3.2).^3^ AcPases are enzymes that are ubiquitous and widely distributed in microorganisms, insects, animals, and plants.^4–9^

Microorganisms are vital in regulating P soil availability and P transformation processes.^10^ Fungi can solubilize insoluble phosphorus by acidification/chelation or phosphatase-mediated hydrolysis of organic phosphate.^11^ *Trichoderma* are ubiquitous and free-living fungi commonly found in soil, decaying wood, and root systems. They are well-known as effective biocontrol agents for several plant pathogens.^12^

*Trichoderma harzianum* is a saprophytic fungus known for its potential as a biological control agent and acts in the release of carbon, nitrogen, and phosphorus into the environment. Phosphorus is released through the action of phosphatases, converting organic phosphate into a soluble inorganic form by hydrolysis.^13^

In the FES process, the required water content is absorbed by the substrate in a solid matrix and offers further advantages for the growth of microorganisms by oxygen transfer.^14^ This technique has proven to be advantageous because, in addition to simulating the natural habitat of wild fungi, it has greater productivity of enzymatic extracts, less susceptibility to inhibition and greater stability of enzymes to variations in temperature and pH.^15^

There has been a growing trend toward using grain-based processes that employ solid-state fermentation for enzyme production. Solid substrate fermentation (SSF) is a process in which the substrate acts as a carbon (and energy) source, occurring in the absence or near-absence of free water. SSF is a promising technology for enzyme production utilizing low-cost and renewable biomass that mimics the natural habitat of microorganisms, particularly fungi.^16^ Marui et al.^17^ evaluated a comparison of acid phosphatase gene expression profiles in solid-state rice and soybean cultures of an *Aspergillus oryzae* strain with low acid phosphatase activity.

Previous studies have shown that *T. harzianum* can produce acid phosphatases with relevant biochemical properties. Leitão et al.^11^ reported the purification and characterization of an extracellular ACPase from *T. harzianum*, with optimal activity at pH 4.8 and 55 °C, broad substrate specificity, and strong inhibition by tungstate. Souza et al.^18^ characterized a new, thermally stable ACPase (ACPase II) with high affinity for various phosphate substrates, suggesting its biotechnological potential. These studies were performed under submerged fermentation conditions using synthetic media.

It is well-known that phosphate plays an important role in enzyme production. For instance, the type and the amount of phosphatase produced by *T. harzianum* depend on the concentration of phosphate in the growth medium.^19^ Therefore, the purpose of this work is to study the effect of phosphate on the fungal biomass, phosphatase production using millet during solid-state fermentation using *T. harzianum*. Although it was found that this microorganism is a good phosphatase producer,^20^ a literature survey showed that there is not much published information on the production and characteristics of phosphatase for this microorganism.

Therefore, the relevance of phosphatases in phosphate solubilization is being considered, as well as increasing soil fertility and plant development. This research evaluates the production of acid phosphatases in solid fermentation using the fungus *Trichoderma harzianum* and millet as substrate.

## 2 Materials and Methods

### 2.1 Microorganism

The fungus *T. harzianum* was isolated in the Chemical Mineral Complex of Araxá (Vale Fertilizantes), Minas Gerais—Brazil. This fungus was identified by the biochemical test of conventional taxonomy, by the André Tosello Research and Technology Foundation (Campinas-SP). This fungal culture was preserved using Castellani’s method in distilled water. The activation of the microorganism was carried out by cultures in Petri dishes containing dextrose agar (PDA) with the composition in g/L: NaNO_3_ 2.0, K_2_HPO_4_ 1.0, MgSO_4_ 0.5, KCl 0.5, FeSO_4_ 0.01, Sucrose 30.0 and Agar 20.0.

### 2.2 Morphological Analysis of *T. harzianum*

The morphological analysis of *T. harzianum* was done using scanning electron microscopy (SEM). The material for the SEM was the grown *T. harzianum* in culture medium. The samples were prepared for analysis according to the modified method.^21^ The modified Karnovsky fixative was prepared with 2.5% glutaraldehyde, 2.5% paraformaldehyde, 0.05 M sodium phosphate buffer at pH 7.2, and 0.001 M CaCl_2_. The samples were dried to the critical point and, finally, metalized with gold to allow for analysis in the SEM.

### 2.3 Determination of Moisture

The moisture (*h*) was determined following eq 1

1 $$h\;\left(\%\right)=\left[\frac{\mathrm{wet}\;\mathrm{sample}\;\mathrm{weight}\;\left(\mathrm{WW}\right)-\mathrm{dry}\;\mathrm{sample}\;\mathrm{weight}\;\left(\mathrm{DW}\right)}{\mathrm{dry}\;\mathrm{sample}\;\mathrm{weight}\;\left(\mathrm{DW}\right)}\right]\times 100$$

where

wetsampleweight(WW)=(waterweight(g)+sampleweight(g))

drysampleweight(DW)=drysampleweight(g)

### 2.4 Determination of Cellular Biomass

Biomass for fungi was determined by filtering crude fermented broth. The paper filters, previously weighed, had a diameter of 90 mm and particle retention of 4–7 μm. After being filtered, the filters with the biomass were taken to an oven at a temperature of 100 ± 1.0 °C until mass constant. The difference in filter mass before and after the over was the mass of biomass in the broth volume fermented. The cell concentration was in (g/L). The filtered volume was reserved for the analysis of acid phosphatase activity.

### 2.5 Determination of pH

The pH was measured with a Gehaka pH meter previously calibrated for each sample.

### 2.6 Acid Phosphatase Activity Assay

According to Leitão et al.,^11^ acid phosphatase activity was measured with modifications. The reaction occurred between the crude enzyme and the substrate 5 mM *p*-nitrophenyl phosphate hexahydrate disodium salt (*p*-NPP) (Sigma-Aldrich) in 50 mM sodium acetate buffer (pH 5.0) at 40 °C. After 15 min, the reaction was stopped by adding 1 mL 0.1 M NaOH. The amount of p-nitrophenol was measured at 405 nm.^20^ A unit (1U) of acid phosphatase activity was defined as 1 μM *p*-nitrophenol (*p*-NP) formed per minute.^22^

### 2.7 Preliminary Experiments: Phosphatase Production Using Cultivation with Distilled Water and Extractions with Tween 80 and Sodium Acetate Buffer

A moist cultivation method in pH 5 and two enzymatic extraction methods one with sodium acetate buffer 0.005 M in pH 5 and the other with 1% Tween 80 in water were used.

#### 2.7.1 Inoculum

Two Petri dishes (100 mm × 20 mm) with *T. harzianum* were scraped and added in 20 mL of sterile distilled water pH 5. These cells were then used in the FES to produce the enzyme complex.

#### 2.7.2 FES

In a static conical flask reactor (500 mL), 20 mL of sterile water at pH 5 was inoculated with *T. harzianum* cells at a concentration of 3 × 10^8^ conidia/mL (4 g/L) and added to 100 g of previously sterilized millet substrate (temperature of 121 °C and 1 atm of pressure for 30 min). In FES a static reactor was discarded for each sample point. The control experiments were carried out for each FES sample point for each substrate without *T. harzianum* cells. The cultivation was in a 150 L ALFA MARE AM5020 BOD incubator at 24 °C.

#### 2.7.3 Enzyme Extraction

The enzymes were extracted using 100 mL of sodium acetate buffer 0.005 M at pH 5 or 1% of Tween 80 in water. After adding the extractive medium, the solid fermented medium was stirred using a glass rod and filtered to obtain the crude enzyme extract.

### 2.8 Central Composite Design

All statistical analyses were performed by Statistica software (Statsoft, Inc.). A central composite design (CCD) method was used to determine the number of experiments to be evaluated for variables, in the optimization responses.

The central composite design (CCD) was constructed with three levels, with three replicates at the central point. It included three variables: millet mass, moisture, and inoculum concentration in two responses ACPase (U/mL) and Biomass (g/L). All experiments were performed in triplicate. The α used was the rotatability, and its absolute value was 1.68 (Table 1).

**Table 1:** Shows the Real and Coded Values of the Analyzed Variables

	level				
variables	–α	–1	0	+1	+α
millet mass—*X*1 (g)	66.36	80	100	120.0	133.63
moisture—*X*2 (%)	16.36	30.0	50	70	83.63
inoculum conc.—*X*3 (g·L^–1^)	1.31	2	3	4	4.68

## 3 Results and Discussion

### 3.1 Morphology of Fungi by SEM

Scanning electron microscopy shows coiled hyphae of *T. harzianum* producing spore-like structures (Figure 1). The surface of *T. harzianum* conidia, which appears smooth under an optical microscope, reveals a rough and verrucose texture when observed at a high magnification of 20,000×.

**Figure 1:**
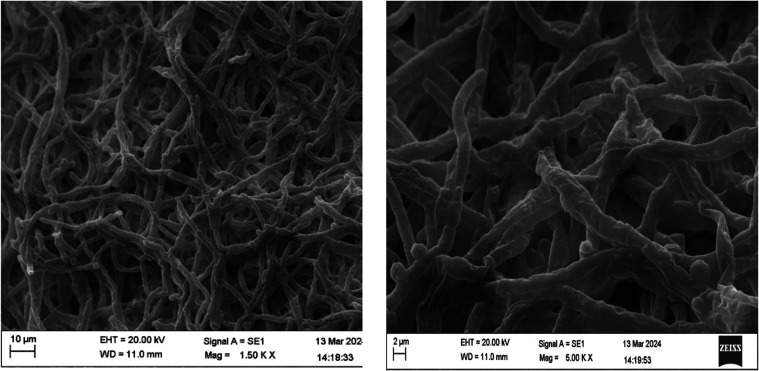
Morphology of the mycelium of *T. harzianum* growing in PDA (CK) medium by scanning electron microscopy.

The surface characteristics of fungal conidia appear rough when examined at high magnification, something not visible with optical microscopy. Additionally, details of hyphae with unidirectional apical growth, capable of dichotomous branching, were frequently observed. Phialospores of several *Trichoderma* species are loosely attached through epispore scars. In the case of *T. harzianum*, for example, the roughness of the spore surface was observed at the electron microscopic level, and this roughness was attributed to intermittent partial thickenings of the outer epispore wall.

### 3.2 Evaluation of the Use of Sodium Acetate Buffer (0.05M) at pH 5 and Tween 80 (1%) in Water for the Extraction of Acid Phosphatase

Table 2 presents the results obtained in the cell growth SSF, pH, and conidia production (conidia/mL) for cultivation in millet with 20% moisture and phosphatase extraction using sodium acetate buffer and Tween 80.

**Table 2:** Results of Phosphatase Production Assays with 20% Moisture in Millet and Extraction of Acid Phosphatase with 0.05 M Sodium Acetate Buffer and Tween 80

extraction with 0.05 M sodium acetate buffer					extraction with Tween 80			
time (days)	biomass (g/L)	pH	conidia (conidia/mL)	AcPase U/mL	biomass (g/L)	pH	conidia (conidia/mL)	AcPase U/mL
0	1.00 ± 0.25	5.00	3.00 × 10^8^	0.00	3.00 ± 0.15	4.00	3.00 × 10^8^	0.00
3	1.13 ± 0.25	5.35	4.50 × 10^8^	5.00	3.61 ± 0.15	5.21	3.90 × 10^8^	9.69
5	1.94 ± 0.25	5.37	5.10 × 10^8^	7.27	5.26 ± 0.15	5.52	5.66 × 10^8^	11.88
7	2.68 ± 0.25	5.41	6.77 × 10^8^	10.87	7.51 ± 0.15	5.08	7.81 × 10^8^	13.64
9	3.15 ± 0.25	5.67	7.13 × 10^8^	11.00	9.68 ± 0.15	5.33	8.26 × 10^8^	18.21

The incubation period is a crucial factor for enzymatic biosynthesis. The maximum production of acid phosphatase reached 18 U/mL with extraction using Tween 80, while with sodium acetate buffer extraction, it was 11 U/mL. The assay in which extraction was performed with Tween 80 showed higher biomass growth and conidia in the same order of magnitude, which could also be responsible for the higher value in acid phosphatase concentration. Zhao et al.^23^ investigated phosphorus solubilization in three phosphate sources: tricalcium phosphate (TCP), dicalcium phosphate (DCP), and phytic acid in the presence and absence of salt. The highest acid phosphatase production was 9.61 × 10^–3^ U/mL for the culture medium containing phytic acid as a P source. In the extraction with sodium acetate buffer (pH 5), a gradual increase in biomass and conidia production was observed, along with a slight rise in phosphatase activity over the days. The biomass increased with time, indicating continuous fungus growth with intensified conidia production. Phosphatase activity was detected only after a few days of cultivation, showing a progressive increase until the end of the experiment. These results align with the literature, where solid-state fermentation systems generally exhibit gradual biomass and enzyme activity growth as the mycelium develops and adapts to the substrate. On the other hand, Tween 80 extraction demonstrated a quicker rise in biomass and higher phosphatase production in a shorter time. This can be attributed to the surfactant’s action, which facilitates the rupture of fungal cells and promotes a release of the enzyme. The significant increase in conidia production also suggests that Tween 80 enhances cell growth and sporulation. The surfactant’s action may have enabled a more efficient interaction between the substrate and the growing environment, resulting in improved conditions for enzyme production. A comparison of the two extraction methods reveals that while sodium acetate buffer effectively maintained the proper pH for phosphatase, Tween 80 proved more efficient at releasing the enzyme and encouraging cell growth and sporulation. These findings corroborate the studies by Velásquez-Quintero et al.,^24^ which highlighted the role of Tween 80 in enhancing cell permeability and optimizing the production of extracellular enzymes, such as laccase, in solid fermentation systems. The literature also indicates that surfactants, like Tween 80, can facilitate the release of enzymes from mycelial systems, improving enzyme recovery.^19^ In the study of Nongpiur et al.,^19^ the use of buffers and surfactants was also crucial for acid phosphatase extraction efficiency, confirming that the choice of extraction medium directly affects enzyme yields.

With 20% moisture, the best production of biomass and conidia reached values of 9.68 g/L and 8.26 × 10^8^ conidia/mL in the assay where extraction was performed with Tween 80, compared to the first extraction method. Initially, there is significant growth in both biomass and conidia for both solid-state fermentations because the exponential growth (log) phase occurs at the beginning of cultivation, during which microorganisms use the available nutrients to multiply. Another factor is the moisture complexed with the solid substrate or present as a thin layer adsorbed on the surface of a substrate or bound within the capillary regions of the substrate, which has a significant effect on fungal conidial induction, metabolite production, and growth kinetics under solid-state fermentation.^25^ Generally, a minimum of 20% moisture level is essential to facilitate nutrient absorption and fungal growth.^26^ A high moisture level can cause pore filling in the substrate, reducing the oxygen mass transfer coefficient and consequently hindering hyphal growth and the desired production of metabolites.^27^

The pH for the fermentation assays remained close to 5 during the 9 days of incubation, indicating low production of organic acids and staying within the optimal range for phosphatase production. To maintain the pH range and avoid enzyme inactivation, a correction to pH 5 was made in the 0.005 M sodium acetate buffer, which was unnecessary in the extraction with Tween 80 since this solution already had a pH close to 5. This indicates that, while pH significantly influences enzyme production, the action of the surfactant in the Tween 80 treatment was sufficient to ensure high phosphatase yields, even with pH variations. Therefore, extraction with Tween 80 was chosen because it did not require pH correction.

Regarding the substrate used, pearl millet with 20% moisture content demonstrated a good balance between biomass growth and conidia production. Its starch-rich composition and moisture retention capacity were significant factors that promoted fungal development. Compared to the substrates used in the studies by Sala et al.,^28^ such as rice husk and beer draff, the millet used in this research exhibited intermediate characteristics. Rice husk, due to its lower biodegradability, supported greater biomass growth but resulted in lower conidia production. Brewery waste, on the other hand, being more biodegradable and richer in organic matter, led to more intense sporulation. Millet, in turn, provides a balance between the attributes of these two substrates, promoting both mycelial growth and sporulation depending on the fermentation purpose. This substrate offers an effective equilibrium between moisture, biodegradability, and nutrient retention, which are essential factors for maximizing both mycelial growth and conidia production. This intensifies the idea that millet is an excellent choice for solid-state fermentation compared to other substrates. Sala et al.^28^ reveals that pearl millet can be a viable option, offering advantages for both biomass growth and sporulation, compared to *rice husk*, which favors growth but limits sporulation, and *brewery waste*, which favors sporulation but may have limitations in terms of robust biomass growth. Millet, with its balanced characteristics, can provide an ideal medium for solid-state fermentation, especially when the goal is to optimize both processes. These results are also corroborated by the findings of Nongpiur et al.,^19^ who emphasized that the choice of substrate has a direct impact not only on biomass but also on spore production, corroborating the importance of selecting a substrate that promotes both processes in a balanced way. These results also indicate that the choice of substrate and extraction method has a direct impact on the efficiency of enzyme production and the quality of the fermentation process. The use of Tween 80 proved to be an effective strategy for the optimization of acid phosphatase extraction, while pearl millet stood out as a balanced substrate, favoring both cell growth and conidia production.

### 3.3 Evaluation of the Combined Influence of Millet Mass, Moisture, and Initial Millet Concentration

After the results of the preliminary tests, it was possible to conduct the Central Composite Design (CCD). The CCD was developed to maximize the acid phosphatase production by the *T. harzianum* strain. This design was proposed with 3 levels, 3 replications at the central point, and 2 variables were studied: *X*1–Millet mass, *X*2–Moisture, and *X*3–Inoculum concentration, totaling 17 treatments. In this design, the rotational α value was 1.68. Statistical experiment design is an efficient approach for optimization, particularly in predicting interactions between variables and identifying significant components affecting phosphatase production.

The responses analyzed were the activity of acid phosphatase (AcPase) expressed in (U/mL) and dry biomass (g/L) in 9 days to the final time of solid-state fermentation are presented in Table 3. All statistical analyses were done using the StatSoft Software Statistica 7.1.

**Table 3:** Central Composite Design with the Studied Variables and Analyzed Responses

experiment	*X*1 (g)	*X*2 (%)	*X*3 (g/L)	ACPase (U/mL)	biomass (g/L)
1	(−1)80.00	(−1)30.00	(−1)2.00	12.24	4.38
2	(−1)80.00	(−1)30.00	(+1)4.00	24.01	6.71
3	(−1)80.00	(+1)70.00	(−1)2.00	17.63	4.52
4	(−1)80.00	(+1)70.00	(+1)4.00	21.80	3.94
5	(+1)120.00	(−1)30.00	(−1)2.00	24.28	5.36
6	(+1)120.00	(−1)30.00	(+1)4.00	26.58	6.20
7	(+1)120.00	(+1)70.00	(−1)2.00	23.31	5.13
8	(+1)120.00	(+1)70.00	(+1)4.00	21.90	4.77
9	(−α)66.36	(0)50.00	(0)3.00	22.01	3.68
10	(+α)133.63	(0) 50.00	(0)3.00	25.25	5.84
11	(0)100.00	(α)16.36	(0)3.00	22.92	8.85
12	(0)100.00	(+α)83.63	(0)3.00	13.30	4.43
13	(0)100.00	(0)50.00	(−α)1.31	22.62	5.14
14	(0)100.00	(0)50.00	(+α)4.68	30.94	7.08
15	(0)100.00	(0)50.00	(0)3.00	36.67	9.29
16	(0)100.00	(0)50.00	(0)3.00	36.56	9.78
17	(0)100.00	(0)50.00	(0)3.00	36.05	9.73

Table 3 shows the results for the CCD matrix, including the enzymatic activity ACPase (U/mL) and the Biomass produced (g/L) for *X*1–Millet mass, *X*2–Moisture, and *X*3–Inoculum concentration. The enzymatic activity ranged from 12.24 to 36.67 U/mL, and Biomass from 3.68 to 9.78%. Souza et al.^13^ described acid phosphatase production by *T. harzianum* strains in a culture medium supplemented with 15 g/L of glucose, without the addition of inorganic phosphate, and with the pH adjusted to 4.0. They achieved the maximum acid phosphatase activity of 14.3 U/mg after 48 h of processing. Lima et al.,^20^ studying the immobilization and production of acid phosphatase by *Trichoderma spp*. in soybean molasses, found acid phosphatase activity ranging from 1.5 to 2.5 U/mL after 48 h. Souza et al.^18^ reported average values of 0.07 U/mL for acid phosphatase activity.

The statistical significance of the model’s parameters was analyzed using the *p*-values obtained by Student’s *t* test at a significance level of 90%. Using the results for ACPase and Biomass produced, after multiple linear regression, eqs 2 and 3 were obtained. The models were presented in their reduced form, given that some variables were not significant. Parameters with a significance level lower than 10% (*p* < 0.10) were considered significant. The coefficients of determination (*R* ^2^) obtained after fitting the experimental data to the models were 0.93 for enzymatic activity and 0.94 for Biomass. These results showed good agreement between the experimental values and those predicted by the models. From the Central Composite Design (CCD), it was possible to generate a model for each response (acid phosphatase activity (AcPase) and dry biomass) as a function of independent variables (millet mass, moisture, and inoculum) using Statistica 7.1.

2 $$\mathrm{ACPase}=36.4617+1.8887X1-{4.6516X1}^{2}-1.36691X2-{6.60028X2}^{2}+2.24048X3-{3.51761X3}^{2}-1.88125X1X3$$

3 $$\mathrm{biomass}=9.632+0.4043X1-{1.8211X1}^{2}-0.8587X2-{1.1547X2}^{2}+0.3963X3-{1.3364X3}^{2}-{0.5137X2}_{2}X3$$

In eq 2, the variables *X*1 and *X*3 showed positive effects, and the variable *X*2 had a negative effect. Thus, increasing the Millet percentage and the inoculum concentration led to higher enzymatic activity (ACPase). For the Biomass (eq 3), *X*2 and *X*3 had a positive effect, while *X*2 had a negative effect. Therefore, an increase in *X*2 decreased the response value, indicating that Biomass was inversely proportional to the moisture content.

Additionally, response surfaces were generated by the Statistica 7.1 software. The analysis of the desirability function in Statistica 7.1, allowed the determination of the best conditions for maximizing the two studied responses. Validation experiments were then conducted under optimized conditions, which fell within the study range of the CCD, following the methodology used in the 17 CCD experiments.

#### 3.3.1 Acid Phosphatase Activity (AcPase)

Table 4 presents the results of the statistical analysis related to the response of acid phosphatase activity (AcPase) expressed in (U/mL) and dry biomass (g/L) on the ninth day, the final time of solid-state fermentation. It is possible to observe in the analysis of ANOVA for acid phosphatase (AcPase) that the values of the tabulated Fisher-Snedecor F distribution (FTab) were lower than the calculated values (FCalc). Therefore, the regressions are considered efficient and can represent the system.

**Table 4:** ANOVA of Factors and Their Interactions for Acid Phosphatase (AcPase)

assuming α = 10%						
source of variation	sum of squares	degrees of freedom	mean square or variance	*F*_calculated_	*F*_tabulated_ (7.9)	*p*-value
regression	764.798	7	109.256	16.629	2.505	1.7 × 10^5^
residuals	59.131	9	6.570			
total	823.929	16				

Analysis of variance (ANOVA) indicated that the model adjusted for acid phosphatase activity (AcPase) is highly significant. The calculated *F* value (16.629) was higher than the critical *F* value (2.505), and the extremely low *p*-value (1.7 × 10^5^), close to zero, was lower than the significance level adopted (α = 1.68), corroborating the relevance of the factors analyzed for the response. The sum of squares of the regression (SQ Regression = 764.798) considerably exceeded the sum of squares of the residuals (SQ Residual = 59.131), suggesting that most of the observed variation can be explained by the adjusted model. These results are consistent with those found by Lima et al.^20^ who also observed very low p-values and highlighted the importance of interactions between variables to explain the variability observed in the data, reinforcing the robustness of the model used in this study.

From the results of the regression ANOVA, it was possible to construct response surfaces and define optimal regions. In Figures 2, 3, and 4, response surfaces and contour plots are presented for the AcPase response as a function of millet mass (*X*1) and moisture (*X*2); millet mass (*X*1) and inoculum concentration (*X*3); and moisture (*X*2) and inoculum concentration (*X*3), respectively.

**Figure 2:**
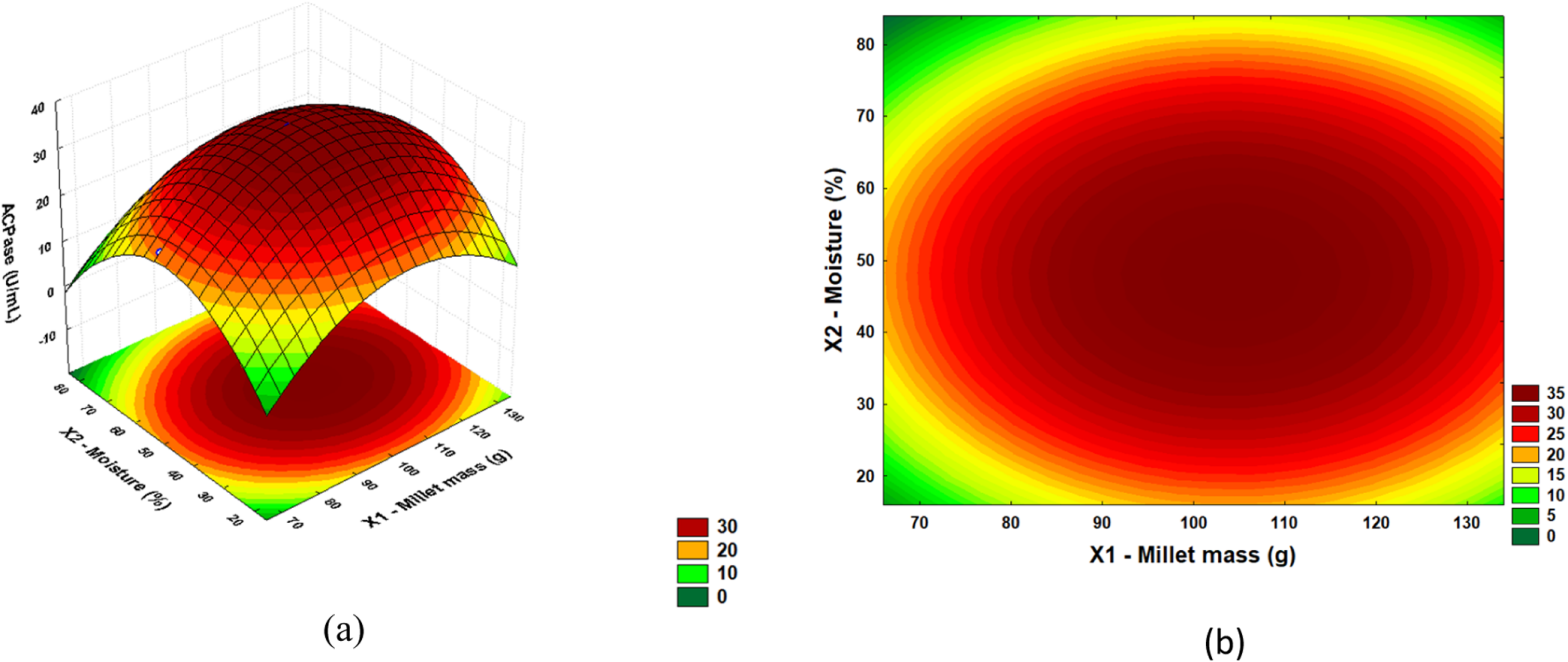
(a) Response surface and (b) contour plot for acid phosphatase activity with moisture (%) and millet mass (g).

**Figure 3:**
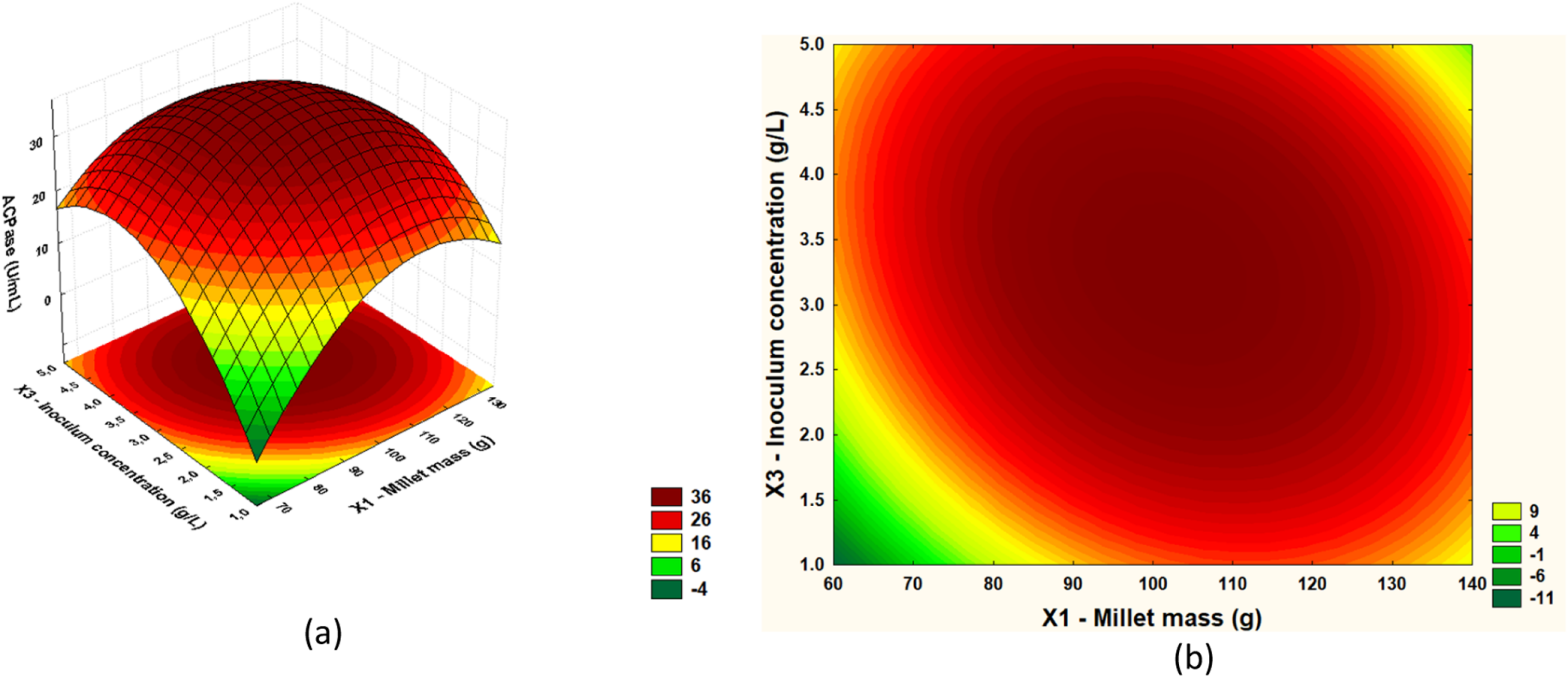
(a) Response surface and (b) contour plot for acid phosphatase activity in relation to inoculum concentration (g/L) and millet mass (g).

**Figure 4:**
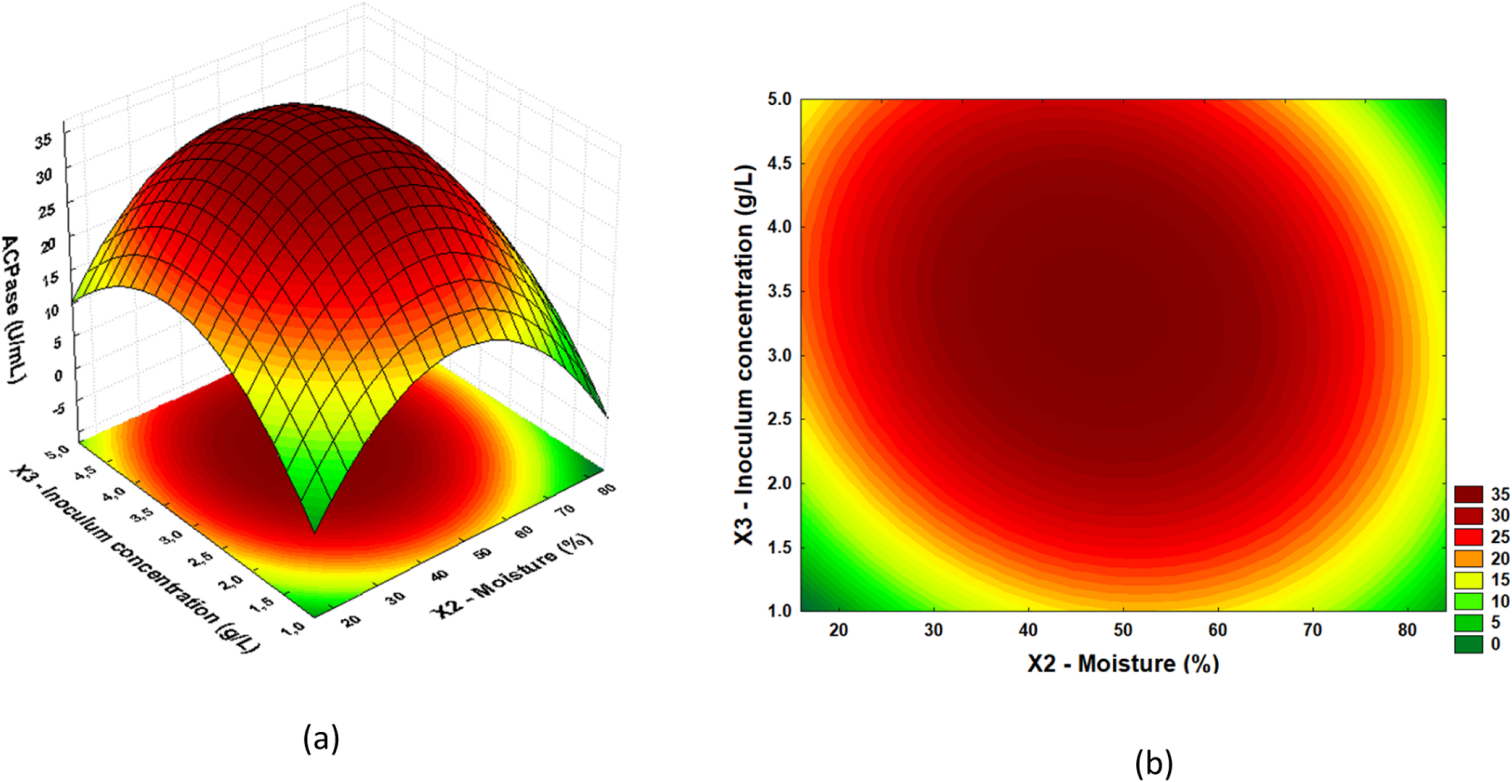
(a) Response surface and (b) contour plot for acid phosphatase activity as a function of inoculum concentration (g/L) and moisture (%).

Figure 2(a),(b) show the interaction between moisture (%) and millet mass (g) for acid phosphatase production. The highest acid phosphatase titers were recorded in a moisture range of 30 to 60 and millet mass of 90 to 120 g. However, the enzyme activity decreases with further increases in moisture and millet mass. Changes in moisture above or below the ideal value can impair microbial growth, reduce enzyme activity, and alter nutrient availability, as well as hinder oxygen diffusion. This can lead to suboptimal conditions for solid fermentation, where acid phosphatase production decreases.

Figure 3(a),(b) show the interaction between inoculum concentration (g/L) and millet mass (g) for acid phosphatase production. The highest acid phosphatase titers were recorded in an inoculum concentration range between 2.5 and 4.5 g/L and a millet mass of 90 to 120 g. However, enzyme activity decreases when inoculum and millet mass concentrations are very low. Reducing these parameters compromises the process, as the insufficient amount of inoculum limits the microbial population required for effective fermentation, which reduces the production of acid phosphatase. In addition, a smaller millet mass may not provide enough nutrients for the proper growth of microorganisms, resulting in reduced fermentative activity.

Figure 4(a),(b) illustrate the interaction between inoculum concentration (g/L) and moisture (%) in acid phosphatase production. The highest acid phosphatase titers were observed in an inoculum concentration range between 2.5 and 4.5 g/L, associated with a millet moisture ranging between 40 and 65%. The decrease in enzyme activity, observed when inoculum concentrations are very low or humidity is outside the ideal range, can be explained by several factors. Insufficient inoculum concentration limits the available microbial population, compromising substrate colonization and acid phosphatase production. In addition, very low humidity reduces the availability of water required for the metabolic reactions of microorganisms, resulting in reduced enzyme activity. On the other hand, excessive humidity can create anaerobic conditions, impairing the growth of aerobic microorganisms and limiting the diffusion of oxygen and nutrients, which negatively affects enzyme production. Thus, both inadequate inoculum concentrations and humidity outside the ideal range result in less efficient fermentation, with lower acid phosphatase production.

#### 3.3.2 Production of Dry Biomass

Table 5 presents the statistical analysis for the response of dry biomass activity (g/L) in 9 days, the final time of solid-state fermentation. It is possible to observe in the ANOVA analysis for dry biomass that the values of the tabulated Fisher-Snedecor F distribution (FTab) were lower than the calculated values (FCalc). Therefore, the regressions are considered efficient and can represent the system.

**Table 5:** ANOVA of Factors and Their Interactions for Dry Biomass

assuming α = 10%						
source of variation	sum of squares	degrees of freedom	mean square or variance	*F*_calculated_	*F*_tabulated_ (7.9)	*p*-value
Regression	64.328	7	9.189 0.490	18.751	2.505	1.09 × 10^5^
Residuals	4.411	9				
Total	68.739	16				

From the results of the regression ANOVA, it was possible to construct response surfaces and define optimal regions. In Figures 2, 3, and 4, response surfaces and contour plots are presented for the AcPase response as a function of millet mass (*X*1) and moisture (*X*2); millet mass (*X*1) and inoculum concentration (*X*3); and moisture (*X*2) and inoculum concentration (*X*3), respectively. In Figures 5, 6, and 7, response surfaces and contour plots are presented for the biomass response as a function of millet mass (*X*1) and moisture (*X*2); millet mass (*X*1) and inoculum concentration (*X*3); and moisture (*X*2) and inoculum concentration (*X*3), respectively.

**Figure 5:**
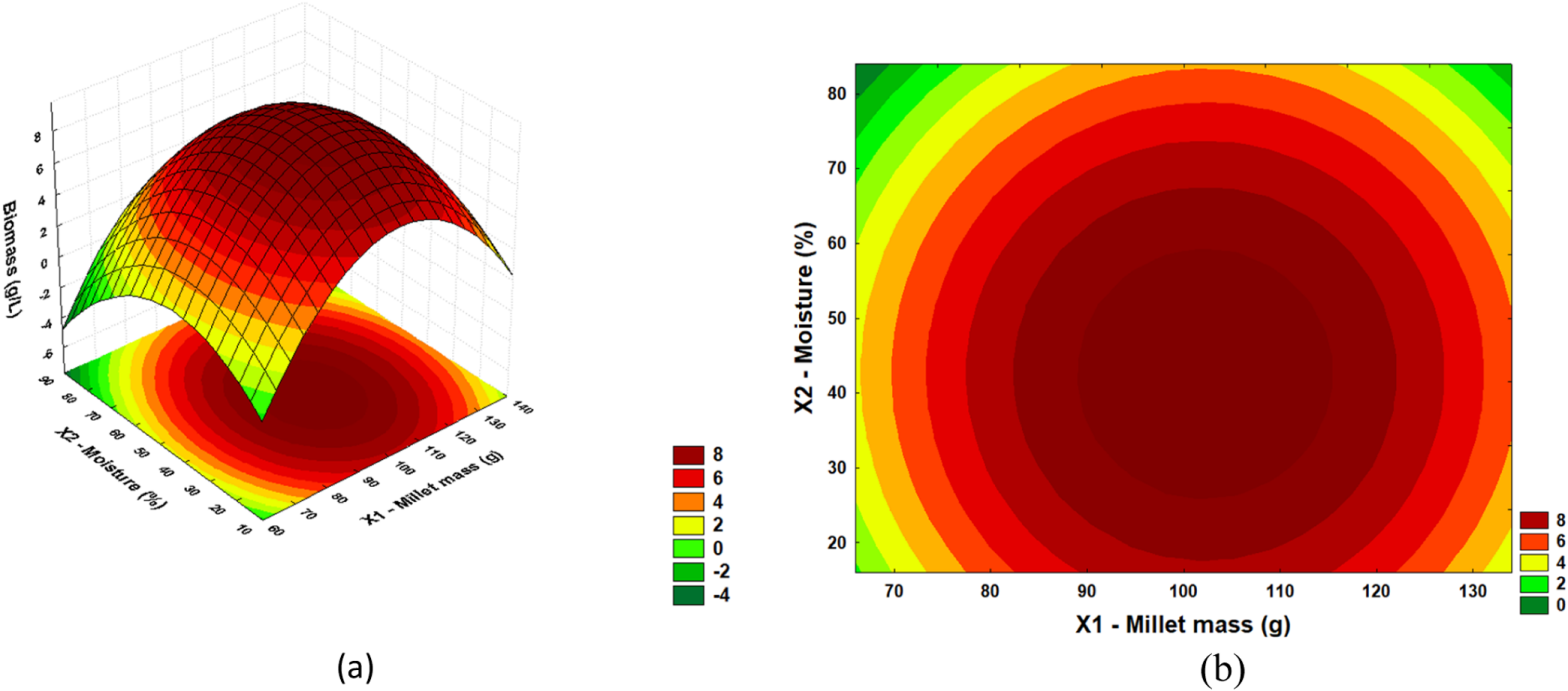
(a) Response surface and (b) contour plot for dry biomass with moisture (%) and millet mass (g).

**Figure 6:**
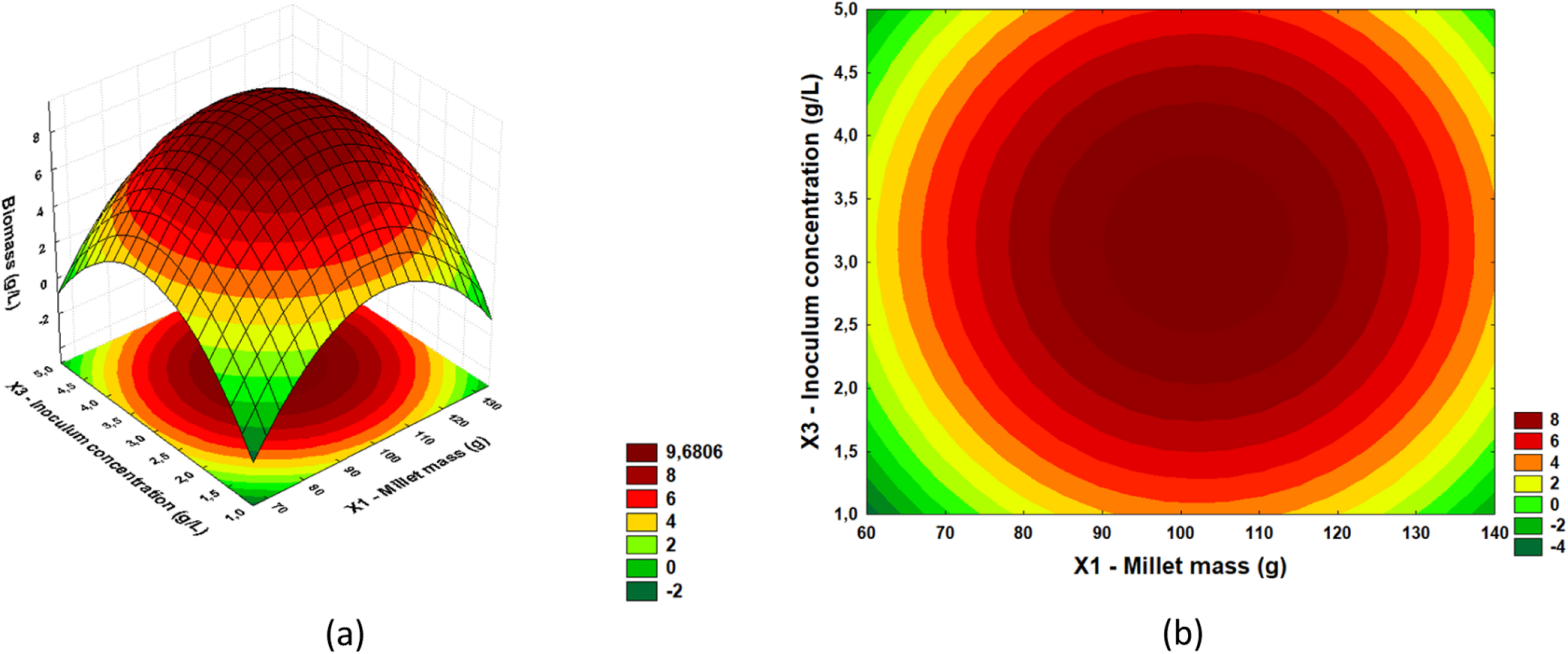
(a) Response surface and (b) contour plot for dry biomass with inoculum (g/L) and millet mass (g).

**Figure 7:**
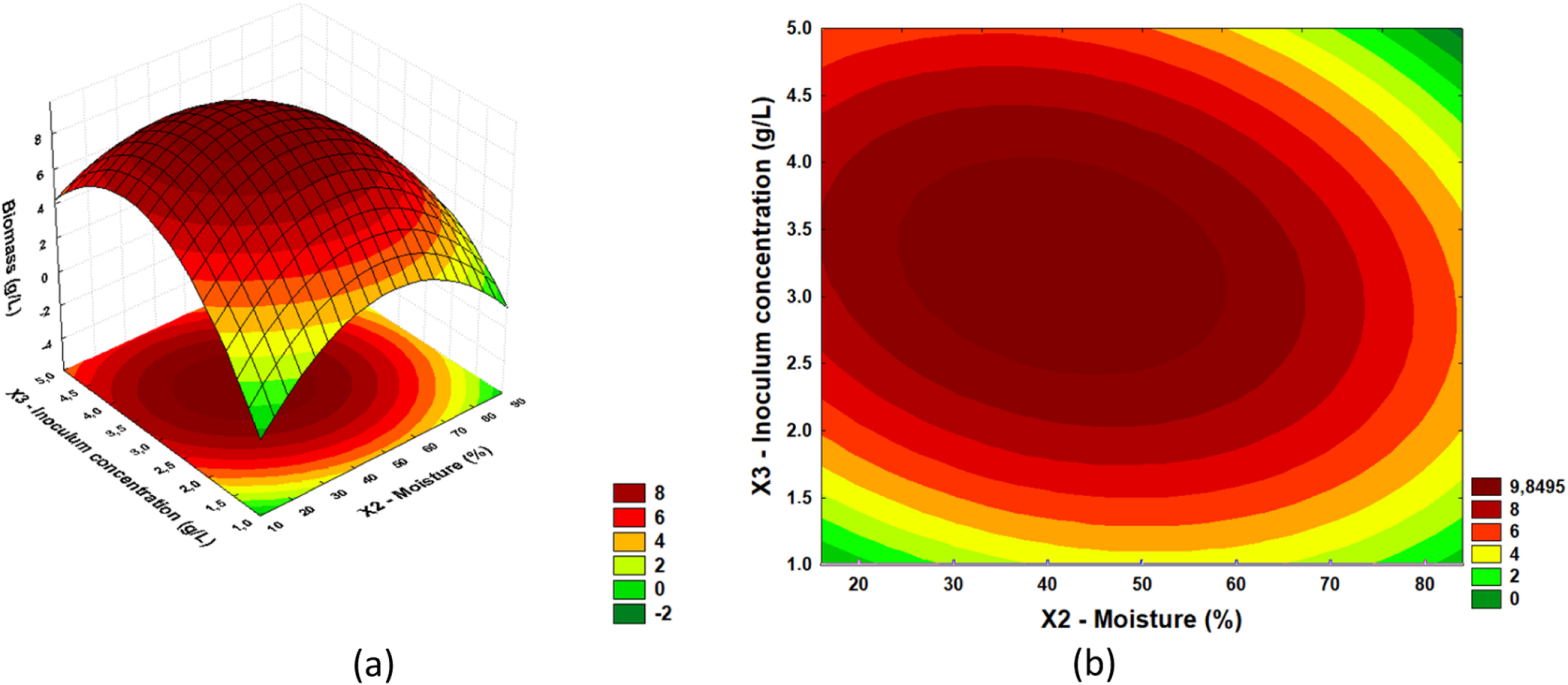
(a) Response surface and (b) contour plot for dry biomass with inoculum (g/L) and moisture (%).

The analysis of the response surface and the contour graphs presented in Figure 5(a),(b) for dry biomass as a function of moisture (%) and pearl millet mass (g) demonstrates the influence of these independent variables on the dry biomass produced. The surface has a curvature, indicating a significant interaction between these factors. The contour plots reveal that the maximum biomass is reached when the millet mass is between 90 and 115 g, and the moisture between 25 and 55%. The response surface and contour graphs presented in Figure 6(a),(b) for dry biomass as a function of inoculum concentration (g/L) and pearl millet mass (g) illustrate a curved surface, suggesting an interaction between these variables. The contour plots indicate that the maximum biomass is obtained with the millet mass between 90 and 115 g and the inoculum concentration between 2.5 and 4.0 g/L. The response surface and the contour plots presented in Figure 7(a),(b) for dry biomass as a function of inoculum concentration (g/L) and moisture (%) show that the maximum biomass is reached when moisture is between 25 and 55% and the inoculum concentration between 2.5 and 4.0 g/L. These results corroborate the study by Zhang et al.,^29^ who investigated the growth of *T. harzianum* T-E5 in agro-industrial residues. Similarly, the contour surfaces presented in this study also showed curvatures, indicating interactions between the variables. Thus, the data obtained reinforces the importance of optimizing experimental conditions to maximize biomass production.

The response surfaces and contour plots for acid phosphatase activity (AcPase) indicated that the region maximizing the production of acid phosphatase suggests millet mass in the range of 90 to 120 g, moisture between 35 and 55%, and inoculum concentration from 2.5 to 4.0 g/L.

For biomass concentration, the analysis of response surfaces and contour plots indicated that to maximize biomass concentration, the millet mass should be between 90 and 115 g, the moisture between 25 and 55%, and the inoculum concentration between 2.5 and 4.0 g/L. Analyzing the intersection of variables for both studied responses, the optimal conditions are a millet mass of 90 to 115 g, moisture between 35 and 55%, and inoculum concentration between 2.5 and 4.0 g/L.

#### 3.3.3 Simultaneous Evaluation of CCD Responses

The determination of the point within the selected ranges by the response surface analysis was carried out using the desirability function in the Statistica program. With desirability, it was possible to determine the condition that simultaneously optimized both the activity of acid phosphatase (AcPase) and biomass concentration, considering the combined influence of the three evaluated independent variables: millet mass, moisture, and inoculum concentration, as shown in Figure 8.

**Figure 8:**
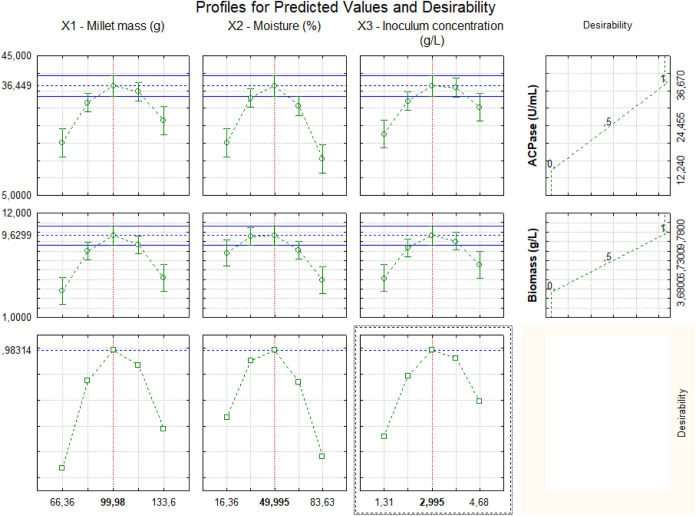
Desirability function profiles for simultaneous maximization of acid phosphatase activity (AcPase) and biomass concentration responses.

According to Figure 8, the top three profiles are the variation in biomass concentration with each factor. The middle three profiles are related to the variation in acid phosphatase activity (AcPase). The fourth profile in these lines presents the acceptable desirability range for maximizing each response (0 ≤ di ≤ 1). The bottom three profiles show the overall desirability, with a value of 0.987. This result indicates a high level of reliability for the application of this simultaneous optimization.

Therefore, the decoded conditions that simultaneously optimize both responses are 100g for millet mass, 50% moisture, and 3 g/L for inoculum concentration. These values are based on the analyses conducted for the optimal ranges of millet mass, moisture, and inoculum concentration, as determined by the results obtained from the response surfaces and contour plots in Figures 2 to 4 and 5 to 7.

After determining this optimal condition (50% moisture; 100 g millet mass; 3 g/L inoculum), it was reproduced experimentally, the pH remained constant and acidic around 4, achieving a maximum production of acid phosphatase of 36.09 ± 0.61 U/mL a value close to that predicted by the model of 36.46 U/mL, the error was 1.03%. For the biomass response, 9.27 ± 0.53 g/L was obtained and predicted by the model of 9.63 g/L (3,91%). Table 6 presents a comparison of acid phosphatase production between this study and similar works reported in the literature.

**Table 6:** ACPase Produced in Research on Phosphatase Production

study	fermentation kind	substrate	extraction	AcPase (U/mL or U/mg)	time (days or hours)	condictions
this research	solid (FES)	millet	Tween 80	36.09 ± 0.61 U/mL	9 days	pH 5, 24 °C
Leitão et al.^11^	submerged	synthetic medium	–	14.3 U/mg	48 h	pH 4.8, 55 °C
Souza et al.^18^	submerged	synthetic medium	–	0.07 U/mL	–	stable to 60 °C
Lima et al.^20^	submerged	soy sauce	–	1.5–2.5 U/mL	48 h	immobilized
Zhao et al.^23^	submerged	phytic acid	–	9.61 × 10^–3^ U/mL	–	salt stress

In Table 6, the solid-state fermentation (SSF) with millet used in this study resulted in significantly higher enzymatic activity (36.09 ± 0.61 U/mL) compared to submerged fermentations using synthetic or agro-industrial media. Leitão et al.^11^ obtained the highest activity among previous works (14.3 U/mg), but under different conditions (submerged fermentation, synthetic medium, and higher temperature). Lima et al.^20^ reported values between 1.5 and 2.5 U/mL using soybean molasses. The superior results achieved here reinforce the potential of solid-state systems and the use of millet as a low-cost, effective substrate for phosphatase production by *T. harzianum*.

Future perspectives include studying the role of acid phosphatase produced by the fungus *T. harzianum* in plant root growth and evaluating the immobilization of phosphatases on active supports to ensure more enzyme stability during application.

## 4 Conclusions

The preliminary tests were crucial in defining specific culture media for the acid phosphatase production and extraction. Using the Central Composite Design, equations for acid phosphatase activity (U/mL) and biomass of the microorganism *T. harzianum* were developed for the variables: millet mass (g), cellular concentration (g/L), and moisture (%). The millet substrate used in solid-state fermentation promoted the production of acid phosphatase with phosphatase activity higher than the values found in the literature, demonstrating its great potential for application.
